# Real-Life Management of Pancreatic Cysts: Simplified Review of Current Guidelines

**DOI:** 10.3390/jcm12124020

**Published:** 2023-06-13

**Authors:** Cătălina Vlăduţ, Dana Bilous, Mihai Ciocîrlan

**Affiliations:** 1 Department 5, “Carol Davila” University of Medicine and Pharmacy, 020021 Bucharest, Romania; catalina.vladut@umfcd.ro (C.V.); mihai.ciocirlan@umfcd.ro (M.C.); 2 Gastroenterology Department, “Prof Dr Agrippa Ionescu” Emergency Hospital, 011356 Bucharest, Romania

**Keywords:** pancreas, cyst, surveillance, diagnosis, endosonography, surveillance

## Abstract

Pancreatic cysts are becoming a popular diagnostic tool due to the increased availability of high-quality cross-sectional imaging. Pancreatic cystic lesions constitute closed, liquid-containing cavities, which are either neoplastic or non-neoplastic. While serous lesions often follow a benign course, mucinous lesions can hide carcinoma and, therefore, require different management. Moreover, all cysts should be considered mucinous until proven otherwise, thus limiting the errors in managing these entities. Due to the need for high contrast soft tissue imaging, magnetic resonance imaging represents an elective, non-invasive diagnostic tool. Endoscopic ultrasound (EUS) has started gaining more prominence with regard to the proper diagnosis and management of pancreatic cysts, offering quality information with minimal risks. Enabling both the acquisition of endoscopic images of the papilla and the endosonographic high-quality evaluation of septae, mural nodules along with the vascular patterns of the lesion contribute to a definitive diagnosis. Moreover, the possibility of obtaining cytological or histological samples could become mandatory in the foreseeable future, allowing for more precise molecular testing. Future research should focus on detecting methods to quickly diagnose high-grade dysplasia or early cancer for patients with pancreatic cysts, thus allowing time for appropriate treatment and avoiding surgical overtreatment or over surveillance in selected cases.

## 1. Introduction

**Figure 1:**
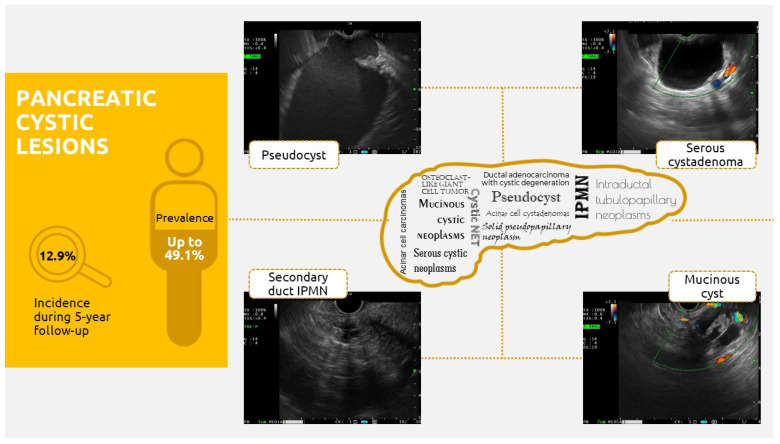
Infographic on pancreatic cystic lesions [4]. The figure was created by the first author; it has not been copyrighted.

Up to 2% of pancreatic cysts represent an incidental finding during CT or MRI for unrelated reasons, which can be grouped into pancreatic cysts that are either neoplastic or non-neoplastic (with their most frequently recognized forms being inflammatory fluid collections). Underlying disorders such as von Hippel–Lindau disease or autosomal dominant polycystic kidney disease (ADPKD) should be included in diagnostic panels since these can be associated with pancreatic cystic lesions [1]. Up to 70% of patients with von Hippel–Lindau disease present pancreatic cysts (serous cystadenomas and cystic pancreatic neuroendocrine tumor) [2]. Studies show that patients with ADPKD present a higher risk of developing pancreatic cysts (36% vs. 23% in patients without ADPKD) [3]. Recent data from the literature show that the prevalence of pancreatic cysts can reach 49.1%, with a 12.9% incidence at a 5-year follow-up, whilst other reports present prevalence ranging from 2.4 to up to 13.5% (Figure 1) [4].

One of the most important steps in this regard is to separate benign from potentially malignant lesions since systematic reviews have shown that the risk of malignant transformation reaches 0.24% per year [5]. The gold standard in diagnosing pancreatic cystic lesions remains magnetic resonance imagining (MRI) combined with magnetic resonance cholangiopancreatography (MRCP). Radiomics can potentially reduce the number of invasive procedures for diagnosing pancreatic cysts by integrating mathematical analysis in cross-sectional imaging. However, endoscopic ultrasonography (EUS) and, in exceptional cases, endoscopic retrograde cholangiopancreatography (ERCP) are useful when requiring tissue (EUS fine-needle aspiration (FNA) or fine-needle biopsy (FNB) or ERCP brushing). Moreover, EUS provides a better characterization of the structure of the cyst and allows one to evaluate the cystic fluid and determine cytology, amylase levels, carcinoembryonic antigen (CEA) levels, and, if possible, diagnostic molecular markers (KRAS, GNAS, VHL, and CTNNB1) and prognostic molecular markers (TP53, PIK3CA, and PTEN), rendering ultrasound endoscopy as a potential essential tool for the proper diagnosis of pancreatic cysts [6,7,8]. Recent studies have shown that positron emission computerized tomography (PET-CT) might also be useful in determining the metabolic activity in a pancreatic cyst, which serves as a marker of malignancy [9]. CT is indicated when there are calcifications within a cyst, thus raising suspicions of a malignant pancreatic cyst and concomitant pancreatic cancer (thus making staging mandatory); when there is suspicion of postoperative recurrence of pancreatic cancer; or for helping differentiate pseudocysts associated with chronic pancreatitis from pancreatic neoplastic cysts [10].

Ensuring the proper management of pancreatic neoplastic epithelial cysts is consistent with the ACG, AGA, and EU guidelines. Conservatory treatment with surveillance imaging is still valid in some cases. Regarding the most frequent pancreatic cysts, surgical treatment is reserved for cysts with positive cytology, worrisome features with respect to malignancy, and cysts with significant malignant potential (e.g., mucinous cystic neoplasms (MCN), main-duct and mixed-type intraductal papillary mucinous neoplasms (IPMNs), and solid pseudopapillary neoplasms (SPNs)). However, more factors, such as age, general wellbeing, and personal choice, must now be taken into consideration, and the risks must always be weighed against the benefits. Alternative treatments such as endoscopic cyst ablation (injection with ethanol or chemotherapeutic agents) are still under examination; however, they are not currently feasible solutions [6,10].

## 2. Where Do We Stand on Pancreatic Cystic Lesions?

Despite numerous studies on pancreatic cystic lesions, only six major guidelines were elaborated by the American Gastroenterology Association in 2015, the American College of Radiology in 2017, the International Association of Pancreatology in 2017, the Revised Fukoka Guidelines in 2017, the American College of Gastroenterology in 2018, and the European Study Group on Cystic Neoplasms in 2018. Not only are there differences between guidelines, but financial burden or resource availability have not been taken into consideration (Table 1) [6,11].

*Pseudocysts* occur secondarily to acute pancreatitis and are non-neoplastic; therefore, the evaluation of previous episodes of acute pancreatitis, whether certified via medical consultation or not, is mandatory. However, one in five people present neoplasia that can lead to acute pancreatitis, rendering accurate diagnosis essential. EUS can help in this regard by adding FNA or FNB, aspirating brown fluid with increased fluid amylase, and low fluid CEA levels [12]. Lymphoepithelial cysts, retention cysts, and “true cysts” remain possible non-neoplastic cystic lesions, though they are rare and still have an unclear etiology.

*Intraductal papillary mucinous neoplasms* (IPMNs) are intraductal epithelial neoplasia composed of a mucin-producing columnar epithelium, and they represent 21–33% of neoplastic cysts. An IPMN presents a potential for malignancy, as it is the most common cystic precursor lesion of pancreatic ductal adenocarcinoma (PDAC) [12]. The gold-standard diagnostic test in this regard is MRI, combined with MRCP. Incorporating a secretin injection improves the visualization of MPD communication in only in 5% of cases. However, there are advantages of EUS when used as a second-line examination tool, including its addition of a contrast-harmonic to observe mural nodules and its facilitation of fluid analysis via FNA (liquid viscosity as a “string sign”; biochemical analysis—high CEA, high amylase, and low glucose levels). Contrast-enhanced EUS offers greater accuracy than MRI or CT in diagnosing a mural nodule. Since 50–60% of fluid samples from IPMNs are acellular, the absence of atypical or malignant cells does not exclude malignancy [13,14]. EUS offers the possibility to obtain tissue and conduct molecular studies on aspects such as GNAS mutation, which is seldom found in pancreatic adenocarcinoma; PanIn-lesions; and MCN. Overexpression of TP53 and SMAD4 and reduced expression of SMARCA4 and RNF43 are associated with worrisome features of an IPMN [15]. Recent studies have shown that high levels of serum mucin-5AC (MUC5AC) levels can help differentiate low-risk from high-risk IPMNs. Cytokine expression in pancreatic cyst fluid is still intensively studied. So far, studies show that higher concentrations of interleukins (IL) 1b, 5, and 8 and prostaglandin E2 (PGE2) are associated with high-grade dysplasia (HGD) or malignancy. Telomere shortening and fusion have been identified in HGD or pancreatic malignancies. MicroRNA profiling via next-generation sequencing (NGS) can be used to determine miRNA expression in pancreatic cancer and pancreatic cysts by compiling a “miRNome” of IPMN cyst fluid in the search for biosignatures of high-risk IPMNs [16,17].

According to the World Health Organization, IPMNs can be classified into the following categories:

- Main-duct IPMNs (MD-IPMNs) (15–21% of IPMNs), which present segmental or diffuse dilatation of the Wirsung duct over 5 mm, without any other cause of obstruction, and are most often localized in the cephalic pancreas. The presence of this lesion is associated with the highest risk of developing malignant features (up to 81%); thus, the corresponding treatment is surgical. Up to 70% of patients are symptomatic [14].
- Branch-duct IPMNs (BD-IPMNs) (41–64% of cases), which are characterized by round cystic lesions that communicate with the pancreatic duct, are most frequently encountered in the pancreatic head (uncinate process), and are often small cysts (5–20 mm) with a grape-like aspect. Indications for the use of a conservative treatment as a “golden standard” with respect to these lesions include multifocality (40% of cases), a high post-surgical recurrence rate (7–8%), and a lower risk of malignant progression (7–42%) [14].
- Mixed-type IPMNs (MT-IPMNs), which present both the above features and are associated with malignant progression in 20–65% of cases. Thus, the corresponding treatment options are the same as those for MD-IPMNs [14].

Treatment consists of the following surgeries: pancreatico-duodenectomy with lymph node dissection (Whipple procedure) in MD-IPMNs and distal pancreatectomy in body and tail MD-IPMN lesions (preferably laparoscopic or robotic). Combining pancreatoscopy with intraoperative intraductal frozen biopsies can guide MPD involvement and the limit of resection. MT-IPMN treatment is the same as that for MD-IPMN. Whilst a BD-IPMN requires surgical treatment only if absolute indications are present, in MD-IPMNs, the indication for surgery remains, even with one relative indication, if the patient does not present significant comorbidities or at least two relative indications, even if the patient presents significant comorbidities. If the cyst is over 2 cm but the patient is young and fit, then surgical treatment should be taken into consideration. For all IPMNs, intraoperative frozen sections of intraductal biopsies are important since these can lead to an oncological extended resection. Concerning multifocal IPMNs, the most suspicious cyst should be removed and total pancreatectomy should be considered only if there are multiple worrisome features or post-surgical recurrence. The overall recurrence rate is approximately 11–20%, reaching 65% in malignant IPMNs [14]. Tumor enucleation was once considered for patients with a non-invasive IPMN, although the risk of post-operative complications including a pancreatic fistula outweighs the benefit; therefore, it is no longer an option. In the case of intrapapilary mucinous carcinoma (IPMC), oncological surgical resection and lymph node dissection are mandatory [17,18]. Whilst HGD and invasive IPMC represent clear indications for surgery, a BD-IPMN with LGD requires monitoring [18]. The survival rates after total pancreatectomy are 80% at 1 year and 65% at 3 years [14,17]. Among patients with invasive ductal adenocarcinoma arising from an IPMN, post-resection adjuvant therapy (chemoradiotherapy) is essential since it improves survival [19].

For patients over 75 years old, surveillance should be individualized based on life expectancy and comorbidities [6]. Surveillance should be lifelong according to EU, Fukuoka and ACG guidelines, except for stable cysts after 5 years and if patients are no longer surgical candidates [12,14]. For patients with relative indication for surgery 6 months follow up is important. The surveillance regime for cysts under 1 cm should be monitoring via MRI every 2 years, annual MRI for cysts measuring 1–2 cm, and MRI/EUS every 6–12 months for cysts measuring 2–3 cm. For cysts over 3 cm, EUS or MRCP are recommended every 6 months for 3 years and then yearly for 4 years [10].

*Mucinous cystic neoplasms* (MCNs) are frequently asymptomatic, with a median age of presentation of 45–48 years. MCNs are most frequent amongst neoplastic cystic lesions, accounting for 10–49% of these lesions. Through CT and MRI, MCNs have been described as solitary cystic lesions with septa (sometimes unilocular) and a thick wall that are situated in the distal pancreas (body or tail) in 95% of cases and do not communicate with and pancreatic duct (as determined via MRCP) [6]. EUS and FNA can offer more data on their macroscopic aspects and enable fluid analysis so that these neoplasms can be distinguished from serous cystadenoma (with low CEA) and IPMNs (with high amylase levels). Up to one third of MCNs are invasive cancers that resemble PDAC and, rarely, colloid carcinoma. Histopathological evaluation is important since 72% of MCNs are benign, 10.5% are borderline neoplasms, 5.5% are carcinoma in situ, and 12% are invasive mucinous cystadenocarcinoma [9]. The corresponding treatment is surgical resection when the cyst diameter surpasses 3 cm, there is an enhancing mural nodule, or the cyst is symptomatic (causing jaundice, acute pancreatitis, and new-onset diabetes mellitus). In lesions under 3 cm, the guidelines for IPMN apply [10].

*Serous cystic neoplasms* are mostly asymptomatic, with a mean age at diagnosis of 62 years. Imagistic diagnosis (CT, MRI, or EUS) is essential, revealing multicystic lesions with a central scar or sunburst calcification (in 20% of cases). The latter is pathognomonic of SCN. Oligocystic lesions or microcystic lesions are rare (10% of cases) and can be misinterpreted as solid lesions through CT. Therefore, EUS is mandatory, revealing a “honeycomb” appearance in microcystic SCN. Moreover, oligocystic SCNs can hardly be differentiated from MCNs or BD-IPMNs; therefore, EUS-FNA and fluid analysis are essential [6]. Cystic CEA levels between 5 and 20 ng/mL are frequently encountered in these lesions [8]. Less than 1% of tumors transform into serous cystadenocarcinoma, for which location in the head and large size are the most frequent risk factors [20]. Management is mainly non-surgical, with imaging surveillance conducted for one year to ensure that rapid growth (5 mm/year) is not present and symptoms or complications do not occur [10].

*Cystic neuroendocrine tumors* (NET) account for 8% of cystic tumors and are most often solitary; non-cephalic; more commonly localized in the neck, body, or tail; non-functional; heterogenous (with respect to solid and fluid content); and discovered in sixth decade of a patient’s life. Imagistic features (CT or preferably MRI) include a peripheral hypervascular rim in the arterial phase that rarely obstructs the Wirsung duct and smooth margins; these features also facilitate the evaluation of liver for metastasis. There is limited information on Octreoscan or Gallium-labeled somatostatin analog positron emission tomography applied to pancreatic NET, which are only useful for poorly differentiated lesions. When visualized using EUS, cystic pNETs present thick walls and septa and allow for the aspiration of the cyst fluid and its analysis [21,22]. The corresponding treatment consists of surgery for lesions over 2 cm, for which tumor location is also considered. For lesions under 2 cm, indolent tumors, and tumors presenting no signs of malignancy, most studies recommend surveillance [10].

*Solid pseudopapillary neoplasms* (SPNs) account for less than 4% of pancreatic cystic tumors and are most often encountered in women under 35 years old. They are localized in the distal pancreas (body or tail) with a heterogenous aspect (solid and fluid component) and rarely present calcifications. Even though SPNs follow a benign course, up to 20% of cases can present with vascular or perineural invasion, lymph node invasion or liver invasion. The corresponding extrapancreatic metastatic sites are the liver, mesocolon, omentum, retroperitoneum, and duodenum [9,20]. Complementary imagistic investigations (CT or MRI) have shown a well-delineated heterogeneous mass (with solid and cystic components) with a peripheral capsule allowing for a more accessible surgical resection. EUS-FNA is often diagnostic, and smaller lesions can only be solid whilst larger lesions present cystic degeneration, sometimes with a hemorrhagic aspect of the aspirate [23]. Treatment is surgery consisting of complete resection and the resection of synchronous or interval metastasis if possible [20].

*Acinar cell cystadenomas* are precursors of acinar cell cystadenocarcinomas. They often result in a benign outcome and were first described in 2000 by Klöppel. They occur more frequently in female patients [6]. Even though MRI and EUS are often non-specific, these techniques can be used to reveal a unilocular or multilocular cystic lesion that seldom presents calcifications, do not communicate with the main pancreatic duct, and can arise in any segment of the pancreas. Even though fluid analyses have shown low CEA and high amylase levels, these cystadenomas are often isolated in small specimen sizes, leading to a false negative result [24,25]. The preferred treatment option remains surgery, which is indicated in symptomatic tumors and for which the risk of malignant transformation and difficulty in making a proper diagnosis prior to surgery are taken into account [26].

*Acinar cell carcinomas* are extremely rare (1% of cases) and present an aggressive, malignant behavior. Acinar cell carcinoma presents particularities: large size with no mass effect on common bile duct and pancreatic duct, an exophytic aspect, and a well-vascularized capsule. Even though over 50% of patients present liver metastasis at diagnosis, the associated prognosis is better than that of PDAC, with a 5-year survival of 72% among patients with resectable disease and 22% for patients with unresectable disease. Treatment consists of surgery of the localized tumor. Synchronous and metachronus liver resection in patients with metastatic disease offers equal survival as that of patients with localized disease [6,9].

*Ductal adenocarcinoma with cystic degeneration* is present in less than 1.6% of cases of PDAC and is more frequently encountered in poorly differentiated, larger PDAC (over 7 cm). Its radiological aspect shows a unilocular, mucin-producing cyst with a peripheral rim of malignant tissue that is eccentrically located, whilst PDAC cystic necrosis is central and often presents hemorrhagic content. These lesions present aggressive behavior, with a higher risk of invading adjacent organs (stomach, kidney, and colon). Treatment of ductal adenocarcinoma with cystic degeneration follows the standard criteria for treatment of ductal adenocarcinoma [6,20].

*Cystic teratomas* (dermoid cyst) are exceedingly rare, slowly growing, benign, germ cell neoplasms. Often present in young patients (10–30 years) with a slight male preponderance, they present as large, heterogeneous lesions containing mature tissue such as sebaceous glands, hair follicles, teeth, and bone [27]. The pancreas is the rarest primary site of a cystic teratoma, while they are most frequently found in the ovaries or testes. Even though the presence of high fat-fluid levels, fat, or calcifications are visually suggestive signs when obtained using abdominal ultrasound, MRI, or CT, these signs can only be encountered in mature teratoma and are thus rare. Therefore, the preoperative diagnosis of a pancreatic dermoid cyst is difficult. Ultrasound endoscopy and FNA with high cytology and CEA evaluations (for which levels higher than 200 ng/mL suggest malignancy) are useful, but they are not conclusive and are associated a risk of seeding in the case of malignancy, especially if a clear surgical indication is present [28].

Treatment: Due to the purely benign nature of mature teratomas, if the diagnosis is well established, then a follow-up is the optimal option. However, due to the difficulty in excluding malignancy and the inaccuracy of cytological specimens, oncological surgical resection is the standard of care [29].

*Intraductal tubulopapillary neoplasms* are rare, slowly growing, premalignant pancreatic lesions that emerge from the pancreatic duct and account for less than 1% of pancreatic exocrine tumors. After the WHO classification issued in 2010, pancreatic intraductal neoplasms can be differentiated into intraductal tubulopapillary neoplasms and IPMNs. Imagistic studies (MRCP, CT, EUS, and ERCP) have shown intraductal solid tumors within a dilated pancreatic duct, which are sometimes associated with pancreatolithiasis due to the insufficient drainage of pancreatic fluid. In 65% of cases, ITPNs are localized in the pancreatic head, in which they always emerge from the main pancreatic duct. Acute pancreatitis can be an initial sign of an ITPN; hence, it should be taken into consideration for patients with idiopathic acute pancreatitis. Several studies have shown that the presence of synchronous or metachronous biliary cancer is suggestive of an ITPN [30]. The corresponding treatment consists of oncological surgical resection, especially due to the malignant potential [31].

*Osteoclast-like giant cell tumors* (OGCT) are extremely rare entities, with an incidence of 1% among all pancreatic tumors, for which fewer than 100 cases have been described. They constitute a relatively aggressive type of neoplasm that is more frequent in females (female:male ratio = 10:1). An OGCT is usually encountered in the sixth to seventh decade of life and involves the body and tail of the pancreas, constituting a cystic-solid lesion. Osteoclastic-like giant cell tumors present a better prognosis than ductal adenocarcinoma, presenting slow metastasis and lymph node spread, whilst pleomorphic giant cell tumors have highly anaplastic malignant potential with early metastasis and poor prognosis [32]. Treatment: Surgery is the first line of treatment and consists of en-bloc resection for lesions under 40 mm with no risk factors [33].

## 3. Guidelines Conundrum on Pancreatic Cystic Lesions—How Can They Be Simplified?

As physicians are encountering this affliction increasingly often, realistic management of these lesions is required. Moreover, atypical and surprising results can be obtained using EUS, which, from our perspective, makes its use essential for any pancreatic cystic lesion. The starting point remains *clinical presentation,* where certain factors are essential, such as a personal history of acute pancreatitis (essential in diagnosing inflammatory cystic lesions), Peutz–Jeghers syndrome, familial adenomatous polyposis syndrome (presenting a higher risk of an IPMN), von Hippel–Lindau (oligocystic SCA is associated with a benign course for 12% of patients), MEN-1 or Wermer syndrome (NET), and a family history of neoplastic pancreatic lesions. Patients can present with abdominal pain, weight loss, acute pancreatitis (25% of cases of IPMN at first presentation), chronic pancreatitis (suggestive of a pseudocyst), jaundice or new-onset diabetes (in malignant tumors), gastric outlet obstruction (nausea and vomiting), recurrent pancreatitis (10% of cases of SCA), an abdominal mass (12% of cases of SCA), hemorrhaging, and even bile duct obstruction (depending on the location of the cyst). Alcohol abuse is a risk factor both for inflammatory lesions and neoplastic ones such as IPMNs [6].

*Blood biomarkers* often present no clinical use for diagnosing high-grade dysplasia or cancer. Even though increased values of CA 19-9 can raise awareness for malignancy in IPMNs, false positive values that can be present in various other afflictions (inflammatory bowel disease, pancreatitis, cirrhosis, cholangitis, benign biliary obstruction, and ovarian cysts) render this biomarker useless in diagnosing cystic pancreatic tumors. Moreover, false negative values can be encountered due to negative Lewis antigen results, which are observed in 10% of the population [34]. Even though changes in α1-3,4 fucosyltransferase metabolism lead to deficient protein fucosylation and false negative CA 19-9 results, almost half of these patients can present increased tumor marker values in PDAC due to partial secretion [35]. Moreover, carcinoembryonic antigen levels or neutrophil-to-lymphocyte ratio have not proven to be effective in diagnosing malignancy [36].

*Imaging methods* can vary from ultrasonography, yielding the lowest accuracy, to MRCP. In current practice, CT is the most frequently used diagnostic tool due to its widespread use. MRCP is the preferred method for the diagnosis and surveillance of pancreatic cystic lesions [10]. Diffuse weight MRI can improve accuracy in diagnosing malignant IPMNs [37]. In both pseudocysts and IPMNs, communication between the pancreatic duct and the cyst can be observed through MRCP or ERCP. For patients who are unfit for surgery or patients without any changes in cyst size or aspect within the last 5 years, there is no need for surveillance. CT or MRI should be sufficient for the initial diagnosis of a pancreatic cyst, but EUS should be used as a complementary tool since it can confirm certain high-risk or worrisome features, thus providing an indication for referral to a multidisciplinary team for evaluation and surgical treatment. MRI remains elective for surveillance among patients with a pancreatic cystic lesion without risk features. However, EUS can be a primary surveillance tool for patients with an MRI contraindication or who have rejected its used and when increased combined surveillance is needed [12,38].

Whilst *EUS* can present specificity similar to that of MRI and CT, the incorporation of different technologies improves the accuracy of differentiating malignant and benign lesions. The possibility of adding contrast to ultrasound endoscopy increases the specificity of this procedure, especially with respect to differentiating non-neoplastic from neoplastic cysts. Whilst CEA cystic fluid cannot be used to differentiate between high-grade dysplasia and malignancy, cytology can offer extensive amounts of information. Despite its high specificity for pancreatic cancer, reaching 90.6% (95 CI, 0.81–0.96), a systematic review and meta-analysis showed a low sensitivity of only 64.8% [39]. Needle-based confocal laser endomicroscopy (nCLE) results have presented a subcellular aspect of these neoplasms, providing in vivo optical biopsies with higher accuracy. Moreover, cystoscopy (Spy Glass) allows for real-time exploration with a fiberoptic probe through a 19G needle, which can be used to search for mucinous lesions and the characteristics of inner epithelial lining. However, both cystoscopy and nCLE are expensive and require centers experienced in pancreatology. EchoBrush was recently introduced, allowing for the harvesting of cystic pancreatic epithelium lining cells during FNA with a 19G needle. Biopsy forceps introduced using a 19G needle is also an option. Both methods are, however, associated with a higher risk of intracystic hemorrhage and are still under evaluation [9,10]. Referring to biomarkers, high values of serum CA 19-9 (over 37 U/mL) are independent factors for malignancy. The diagnostic potential of CEA with respect to differentiating between mucinous and non-mucinous cystic lesions has increased sensitivity and specificity, with values over 192 ng/mL being highly indicative of mucinous cysts [14]. Cystic fluid amylase levels under 250 U/L should exclude the existence of a pancreatic pseudocyst [10]. However, elevated amylase levels within the cystic fluid can be observed in mucinous cysts [40]. Prophylactic antibiotic therapy is mandatory prior to the procedure and for 3 days after the procedure. Despite the risk for tendinitis, Ciprofloxacin is still used for antibiotic prophylaxis, which is administered via the following regime: 500 mg administered orally 60 to 90 min before the procedure or 400 mg iv given over 60 min starting within 120 minutes prior to the procedure). The relative contraindications of EUS-FNA are as follows: over 10 mm of space between the cyst and the transducer and a high risk of bleeding due to a bleeding disorder, antiplatelets, or the personal use of anticoagulation [41,42,43].

*ERCP* offers the possibility to determine cytology via aspiration or brushing, and even pancreatic duct lavage and therapeutic maneuvers, through clearing mucin from the pancreatic duct. However due to the nature of the procedure and the higher sensitivity and specificity of non-invasive methods, it is no longer recommended for diagnosis. Pancreatoscopy offers direct visualization and directed tissue sampling, providing a high accuracy in distinguishing benign from malignant lesions. It can also be used intraoperatively or combined with ERCP, but potential adverse reactions must be taken into account. Malignancy is indicated when observing spotty or linear red markings or vegetative or villous proliferations [6].

**Figure 2:**
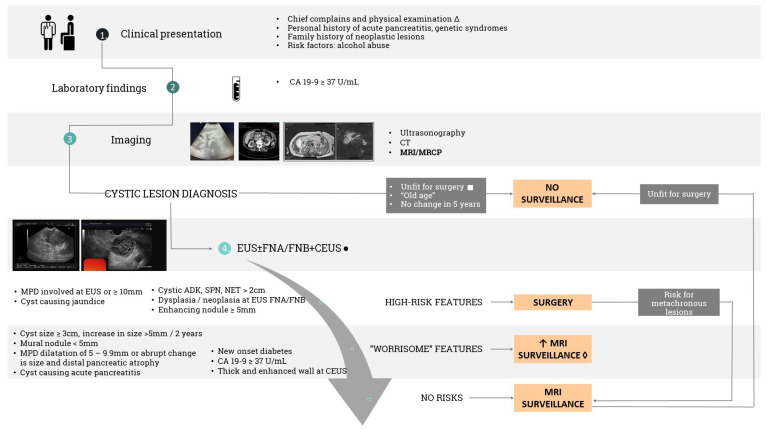
Simplified management of pancreatic cystic lesions [6,20,43,44]. ∆ Abdominal pain, weight loss, acute pancreatitis, abdominal mass (12% of cases of SCA), hemorrhage, and even bile duct and gastric outlet obstruction. ● CEUS is essential in verifying the blood supply both in mural nodules and in thickened walls of pancreatic cysts. ◊ If “worrisome features” are encountered, we recommend increasing the use of combined MRI and EUS surveillance, alternating these 2 investigations at the 3- to 6-month mark or conducting surgery on a case-by-case basis. □ Charlson comorbidity index and Adult Comorbidity Evaluation 27 are scores that aim to select patients that are not medically fit for surgery or will not benefit from surgery and, therefore, do not require further surveillance. The figure was created by the first author and is not copyrighted.

Whilst the revised Fukoka guidelines helped physicians to manage pancreatic cysts, the revised European Experts Consensus statement (issued in 2018) is currently the main reference as we can observe a trend in surveillance rather than surgical resection with respect to IPMNs. Concerning high-risk features, the indication for surgery is absolute, even though EUS can be crucial in establishing some of these features (e.g., dysplasia/neoplasia, enhancing nodule ≥5 mm, etc.). However, relative surgical indications (worrisome features) represent an indication for EUS evaluation, contrast-enhanced harmonic EUS (CEUS), and, if needed, FNA/FNB [14]. A more simplified strategy of managing pancreatic cysts should be globally implemented, especially since resources might vary in different practices (Figure 2).

Despite these recommendations, there are many cases that present atypical findings, raising the question of whether EUS should become essential for the proper diagnosis and further management of pancreatic cysts. These suggestions were also mentioned in a study by Megibow et al., wherein a broad use of EUS was recommended for the better characterization of pancreatic cysts [40]. A contrast-enhanced cystic thickened wall, an increase in size over 5 mm/2 years, and changes in the pancreatic duct diameter are worrisome features according to the Fukoka guidelines, which are still applied by many medical practices to establish the appropriate management regime, though these are not present in the revised EU guidelines from 2018 [6]. Moreover, atypical lymphadenopaties require EUS evaluation with FNB and can thus be considered by some authors to constitute a “worrisome feature” [44].

## 4. Conclusions

Pancreatic cysts are becoming increasingly common in gastroenterology, and physicians can now encounter difficulties in choosing the proper method of dealing with this disease. Endosonography can offer crucial information and sometimes even optimize the treatment of pancreatic cysts, making it crucial in the first diagnosis. There is a need to determine the optimal signature of the cystic fluid since the accessibility of the fluid is much higher; therefore, optimal breakthroughs in the early diagnosis of premalignant lesions is a new hot-topic in pancreatology.

## Figures and Tables

**Table 1:** Cystic lesion management according to 6 major guidelines. 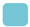 Absolute surgical indication; 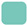 Relative surgical indication; 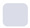 EUS ± FNA indication.

Guidelines	AGA 2015	ACR 2017	IAP 2017	Revised Fukoka 2017	ACG 2018	EAP 2018
SCA, SPN, NET, cystic ADK	SPN, NET, cystic ADK	SCA-symptomatic or >40 mm SPN, NET, cystic ADK		IPMN	SPN	
Jaundice		Present	Present	Present	Present	Present
Acute pancreatitis				Yes	Yes	Yes
New onset diabetes					Yes + MCN/IPMN	Yes
CA 19-9			Elevated	Elevated	Elevated	Elevated
Lymph nodes			Increased	Increased		
Size	>30 mm	>30 mm	>30 mm	≥30 mm	>30 mm	>40 mm
Growth rate	Increasing	+20% longest axe	>5 mm/2 years	≥5 mm/2 years	>3 mm/year	>5 mm/year
Cyst wall		Thickened	Thickened	Thickened		
Nodule	Nodule + MPD dilation	Non enhancing	Enhancing, <5 mm	Enhancing, <5 mm	Yes, at EUS	Enhancing, <5 mm
		Enhancing	Enhancing, >5 mm	Enhancing, ≥5 mm		Enhancing, > 5 mm Solid mass
MPD	Dilated	7–9 mm	Abrupt change with distal atrophy; 5–9 mm	Abrupt change with distal atrophy; 5–9 mm	>5 mm obstructed	5–9 mm
		>10 mm	>10 mm	≥10 mm	Involved at EUS	>10 mm
Dysplasia/neoplasia at EUS FNA cytology	Yes	Yes	Yes	Yes	Yes	Yes

## Data Availability

No new data were created or analyzed in this study. Data sharing is not applicable to this article.
